# Slc15a1 is involved in the transport of synthetic F5-peptide into the seminiferous epithelium in adult rat testes

**DOI:** 10.1038/srep16271

**Published:** 2015-11-05

**Authors:** Linlin Su, Yufei Zhang, Yan C. Cheng, Will M. Lee, Keping Ye, Dahai Hu

**Affiliations:** 1 Department of Burns and Cutaneous Surgery, Xijing Hospital, the Fourth Military Medical University, Xi’an, Shaanxi 710032, China; 2 Heilongjiang Key Laboratory of Anti-fibrosis Biotherapy, Mudanjiang Medical University, Mudanjiang, Heilongjiang 157011, China; 3 The Mary M. Wohlford Laboratory for Male Contraceptive Research, Population Council, Center for Biomedical Research, New York, NY 10065, USA; 4 School of Biological Sciences, University of Hong Kong, Hong Kong SAR, China; 5 Department of Anesthesiology, Taizhou First People’s Hospital, Taizhou, Zhejiang 318020, China

## Abstract

Spermiation and BTB restructuring, two critical cellular events that occur across seminiferous epithelium in mammalian testis during spermatogenesis, are tightly coordinated by biologically active peptides released from laminin chains. Our earlier study reported that F5-peptide, synthesized based on a stretch of 50 amino acids within laminin-γ3 domain IV, could reversibly induce the impairment of spermatogenesis, disruption of BTB integrity, and germ cell loss, and thus is a promising male contraceptive. However, how F5-peptide when administered intratesticularly enters seminiferous tubules and exerts effects beyond BTB is currently unknown. Here we demonstrated that Slc15a1, a peptide transporter also known as Pept1, was predominantly present in peritubular myoid cells, interstitial Leydig cells, vascular endothelial cells and germ cells, while absent in Sertoli cells or BTB site. The steady-state protein level of Slc15a1 in adult rat testis was not affected by F5-peptide treatment. Knockdown of Slc15a1 by *in vivo* RNAi in rat testis was shown to prevent F5-peptide induced disruptive effects on spermatogenesis. This study suggests that Slc15a1 is involved in the transport of synthetic F5-peptide into seminiferous epithelium, and thus Slc15a1 is a novel target in testis that could be genetically modified to improve the bioavailability of F5-peptide as a prospective male contraceptive.

During spermatogenesis in the mammalian testis, two key cellular events, namely spermiation and BTB restructuring, that occur at the opposite ends of the seminiferous epithelium are coordinated by biologically active laminin peptides^1^. Studies from other epithelia have also demonstrated that laminin fragments are biologically active, capable of altering cellular function, including cell movement and cell adhesion^2^,^3^,^4^,^5^,^6^. Our recent study has identified such a laminin-γ3-derived fragment designed as F5 that can induce the impairment of spermatogenesis^7^. Local administration of the synthetic peptide F5 which is composed of 50 amino acids into rat testes was found to cause BTB disruption and germ cell loss^7^, suggesting this laminin peptide fragment may serve as a contraceptive in male rats. However, as the synthetic F5-peptide was administered via direct intratesticular injection, the way how it enters seminiferous tubules and plays further roles beyond the BTB remains unknown.

Peptides are essential in nutrition, and certain peptides serve as important signaling molecules^8^,^9^,^10^. The entrance of peptides into cells/tissues often requires transporter-mediated processes^11^. Slc15a1 (solute carrier family 15, member 1), also known as the peptide transporter 1 (Pept1), is predominantly expressed in small intestine^12^, testis, kidney, liver and brain^13^, and is believed to play key roles in intestinal absorption and renal reabsorption of di- and tri-peptides^14^. As a low-affinity, high-capacity transporter, Slc15a1 has broad substrate requirement and tolerates diverse chemical modification, and therefore accepts various chemicals as substrates^14^. In addition to the transport of endogenous peptides, Slc15a1 also transports exogenous synthetic peptides and various peptide-like drugs, including β-lactam antibiotics, angiotensin converting enzyme (ACE) inhibitors, renin inhibitors, thrombin inhibitors, and the aminopeptidase inhibitor bestatin^15^. Slc15a1 is shown to be a promising target for facilitating intestinal absorption of prodrugs^16^, thus we are interested to know whether Slc15a1 is also involved in the transport of F5, a 50-amino acid polypeptide and potential male contraceptive, into the seminiferous epithelium.

We here report the demonstration of Slc15a1 as a potential carrier for the synthetic F5-peptide. In the present study, the cellular expression and localization of Slc15a1 in rat testes have been investigated for the first time. Local knockdown of Slc15a1 in rat testes by *in vivo* RNAi has shown to impede F5-peptide induced impairment of spermatogenesis, germ cell loss, and BTB disruption.

## Results

### Slc15a1 exists in adult rat testes, germ cells, peritubular myoid cells and interstitial Leydig cells, but not in Sertoli cells

**Figure 1:**
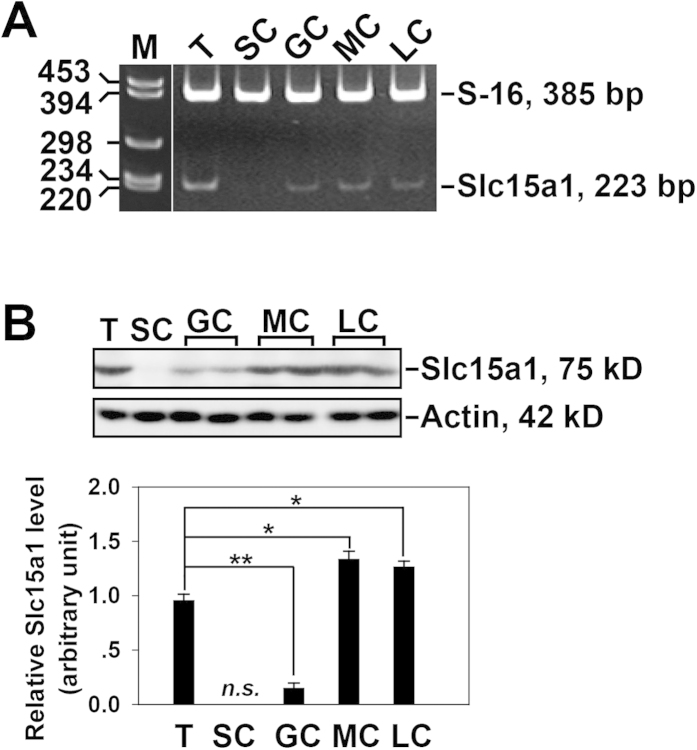
Expression of Slc15a1 in adult rat testes. (**A**) Gene expression of Slc15a1 in adult rat testes (T), Sertoli cells (SC), germ cells (GC), peritubular myoid cells (MC), and interstitial Leydig cells (LC) was analyzed by RT-PCR. S-16 served as a control. M, DNA ladder marker. (**B**) Immunoblotting of Slc15a1 in lysates of T, SC, GC, MC, and LC (∼50 μg proteins/lane) with actin as a loading control. Histogram summarizes the immunoblot results after normalizing each data point against corresponding actin. Slc15a1 protein level in the testis was arbitrarily set as 1. Bars represent means ± sd, *n* = 3. ^*^P < 0.05, ^**^P < 0.01. *n.s.*, not detectable.

The expression of Slc15a1 in various organs including the testes was demonstrated by RT-PCR as described previously^12^,^13^. The current study further explored the cellular expression of Slc15a1 in testes using Slc15a1-specific primer pair as shown in Table 1. The transcript of Slc15a1 was detected in adult rat testes, germ cells, myoid cells and Leydig cells, while not detectable in Sertoli cells (Fig. 1A). The protein level of Slc15a1 was ~5-fold higher in lysates of testes than that in germ cells, and ~1.3-fold higher in myoid cells or Leydig cells than that in testes, while absent in Sertoli cells (Fig. 1B) when assessed by Western blot with a specific anti-Slc15a1 antibody as shown in Table 2. These results corroborated reports in the literature that testis is equipped with Slc15a1^12^,^13^.

### Slc15a1 locates at tunica propria, interstitium, blood vessel endothelia and germ cell nuclei in rat testes

**Figure 2:**
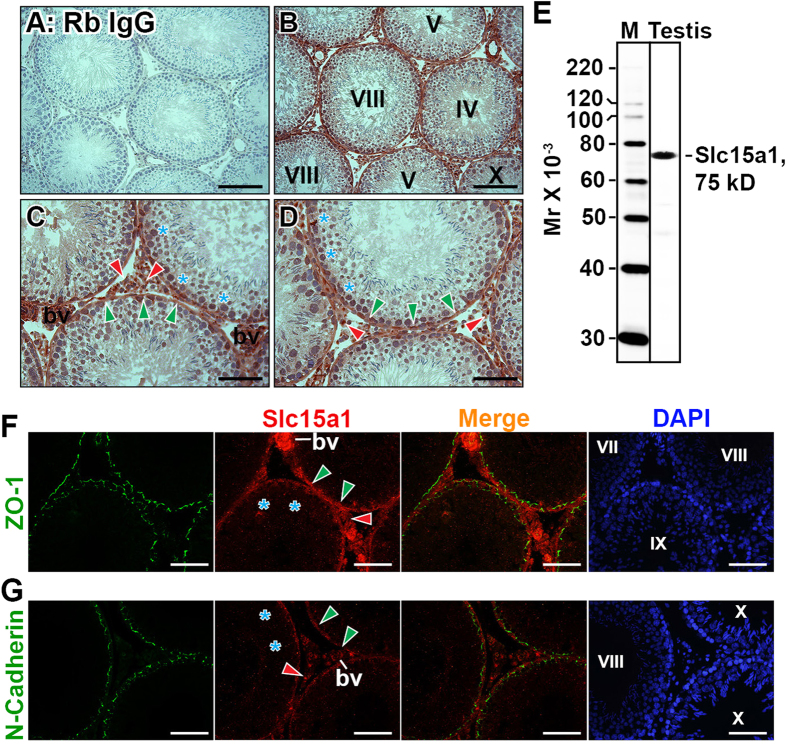
Cellular localization of Slc15a1 in the seminiferous epithelium of adult rat testes. (**A**) Immunohistochemistry was performed using frozen testis sections. Primary antibody was replaced with normal rabbit IgG as negative control. (**B–D**) Specific staining for Slc15a1 appears as brownish-red precipitates. *Green* arrowheads point to peritubular myoid cells, *red* arrowheads point to interstitial Leydig cells, *blue* asterisks indicate Slc15a1-stained nuclei of germ cells, ‘bv’ represents blood vessel. Roman numerals indicate tubule stages. Nuclei were stained with hematoxylin. Scale bars = 150 μm in (**A**,**B**), 100 μm in (**C**,**D**). (**E**) Specificity of the anti-Slc15a1 antibody as demonstrated by an immunoblot of testis lysate (∼50 μg protein). M, protein ladder marker. (**F,G**) Immunofluorescent microscopy was performed on frozen testis sections. Co-localization of Slc15a1 (*red*) with tight junction (TJ) marker ZO-1 (*green*) or adhesion junction (AJ) marker N-cadherin (*green*) in the adult rat testis was shown in **F** and **G**, respectively. Corresponding merged images were also shown, but no area of co-localization was observed (no *orange* color). Roman numerals indicate tubule stages. Nuclei were stained with DAPI (*blue*). Scale bars = 100 μm. Experiments were repeated three times using testes from three rats.

Enzyme immunohistochemistry revealed that Slc15a1 neither located to the site of BTB at all stages of seminiferous epithelial cycle in adult rat testes, nor to the lumenal edge surrounding elongated spermatids at stages VII-VIII at the site of apical ectoplasmic specialization [apical ES] (Fig. 2B–D), two critical ultrastructures securing normal spermatogenesis. Instead, strong Slc15a1 immunoreactivity was detected outside of the seminiferous epithelium at tunica propria (*i.e.*, peritubular myoid cells, noted by green arrowheads), interstitium (i.e., Leydig cells, noted by red arrowheads) and blood vessel endothelium (Fig. 2B–D). A significantly weaker signal was also observed in the nuclei of germ cells (Fig. 2B–D, noted with blue asterisks). Incubating sections with normal rabbit IgG at the same dilution as used for Slc15a1 IgG yielded no observable staining (Fig. 2A). The monospecificity of Slc15a1 antibody was assessed by immunoblotting and a single 75 kD protein band was observed in testis lysates (Fig. 2E), illustrating that this antibody is suitable for enzyme immunohistochemistry and immunofluorescent microscopy experiments. Immunofluorescent staining on adult rat testes revealed that Slc15a1 failed to colocate with BTB tight junction (TJ) marker ZO-1 (Fig. 2F) or adhesion junction (AJ) marker N-cadherin (Fig. 2G), further supporting its absence at the BTB. Instead, the fluorescence signal of Slc15a1 was detected beneath the BTB at tunica propria, interstitium and blood vessel endothelia, and also distributed as scattered spots at the nuclei of germ cells (Fig. 2F,G).

### Local administration of synthetic F5-peptide intratesticularly does not affect the steady-state level of Slc15a1 in adult rat testes

**Figure 3:**
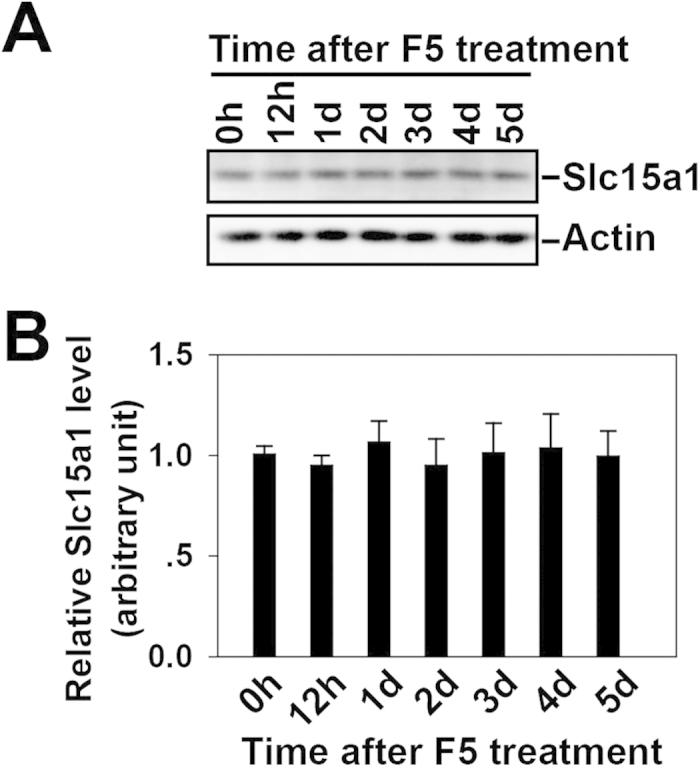
Changes in the steady-state level of Slc15a1 in F5-peptide treated rat testes. Adult rat testes were topically injected with a synthetic peptide F5 to induce the impairment of spermatogenesis. (**A**) Immunoblots of Slc15a1 in 0 h, 12 h, 1 d, 2 d, 3 d, 4 d and 5 d testis lysates following F5 treatment, with actin as a loading control. (**B**) Histogram summarizing results after normalizing each data point against actin. The value at 0 h was arbitrarily set as 1. Bars are means ± sd; *n* = 3. 1-way ANOVA followed by Dunnett’s test.

A previously established *in vivo* model for spermatogenesis impairment was used^7^ to correlate the function of Slc15a1 and peptide transport in the testis. Treating adult rat testis with the synthetic F5-peptide was found to cause reversible BTB disruption and germ cell loss^7^. Slc15a1 is known as an oligopeptide transporter and responsible for peptide absorption in the small intestine^17^. Thus, we were interested to know whether Slc15a1 was also involved in the transport of F5-peptide into the seminiferous tubules in rat testis. We examined the steady-state level of Slc15a1 in adult rat testis after F5-peptide treatment by immunoblotting (Fig. 3A), and found that F5-peptide did not cause the level change of Slc15a1 up to 5 days post-treatment (Fig. 3B).

### The steady-state level of Slc15a1 is effectively inhibited by *in vivo* RNAi in rat testes

**Figure 4:**
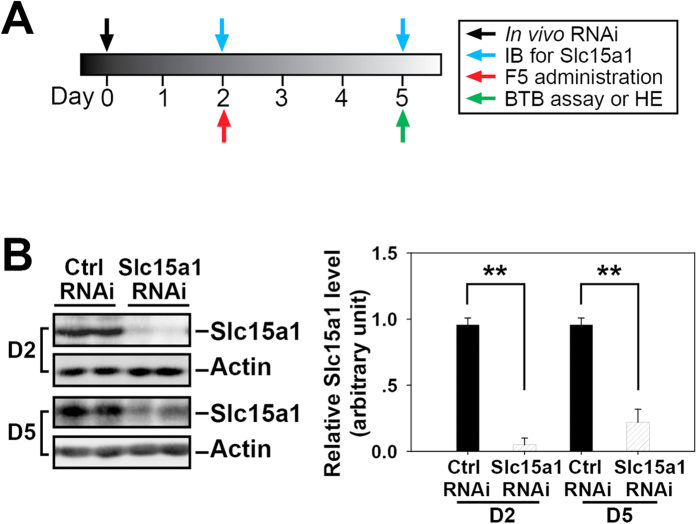
The *in vivo* RNAi of Slc15a1 in adult rat testes. (**A**) The regimen used in this study. Briefly, the right testes of rats (*n* = 9) were injected with 100 nM Slc15a1 siRNA duplexes, while the left ones were treated with 100 nM non-targeting control siRNA on day 0. Two days later (day 2), 3 rats were killed and their testes were removed and lysed for immunoblotting to examine the silencing effect of Slc15a1 RNAi. F5-peptide at 320 μg/testis was then injected to each testis of the remaining 6 rats. Three days thereafter (day 5), 3 rats were sacrificed and their testes were harvested for immunoblotting and HE staining, while the remaining 3 rats were subject to BTB assay. (**B**) Representative immunoblots illustrating an ∼90% and ∼ 75% knockdown of Slc15a1 in the testes on day 2 and day 5 respectively after transfection with siRNA duplexes. ***C***) Densitometric analysis of Slc15a1 immunoblotting data normalized against actin with the control arbitrarily set at 1. Each bar is the mean ± SD of three experiments, ^**^P < 0.01.

To demonstrate the involvement of Slc15a1 in transporting F5-peptide that further induces the impairment of spermatogenesis, we performed *in vivo* RNAi on day 0 to specifically knockdown Slc15a1 expression in rat testes (*n* = 9) (Fig. 4A). Two days thereafter (day 2), testes from 3 rats were removed and lysed for immunoblotting, results showed an ~90% decrease in the protein level of Slc15a1 after RNAi (Fig. 4B). F5-peptide at 320 μg/testis was then immediately administered to the remaining 6 rats via intratesticular injection following the confirmation of effective Slc15a1 knockdown. Three days thereafter (day 5), testes from 3 rats were harvested for immunoblotting to examine Slc15a1 level or HE staining, or used for BTB integrity assay (*n* = 3) in the following experiments. Importantly, lysates from day-5 testes still showed a 75% reduction on Slc15a1 level, demonstrating a relatively stable gene knockdown by *in vivo* RNAi (Fig. 4B).

### Slc15a1 knockdown prevents F5-peptide induced disruption of BTB integrity *in vivo*

**Figure 5:**
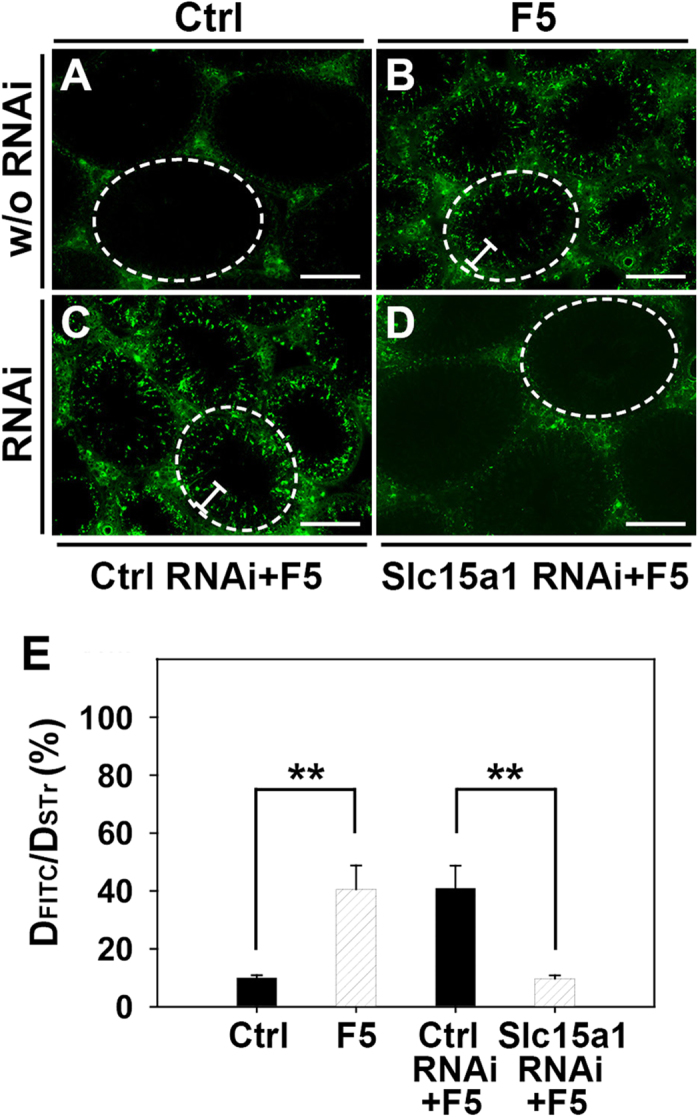
Effect of Slc15a1 knockdown on F5-peptide induced disruption of BTB integrity in rat testes. BTB integrity assay was performed to assess the ability of an intact BTB to block the movement of FITC-inulin across the BTB from the basal compartment near the basement membrane (annotated by “white” dotted line) to the adluminal compartment. Results of the BTB integrity assay were shown in normal testis (Ctrl, **A**), testis treated with F5-peptide at 320 μg/testis (F5, **B**), or testis pre-transfected with non-targeting control siRNA (Ctrl RNAi+F5, **C**) or Slc15a1 siRNA (Slc15a1 RNAi+F5, **D**). Scale bar = 150 μm. (**E**) Histogram summarizing data based on findings shown in (**A–D**) by comparing the distance of FITC-inulin diffused into the epithelium (D_*FITC*_) (annotated by the “white bracket”) *vs.* the radius of a seminiferous tubule (D_*STr*_) (the average of the longest and shortest semi-axes for sections of oval-shaped tubules) (n = 200 tubules from testes of 3 rats in each group). ^**^P < 0.01.

We next sought to examine the role of Slc15a1 in F5-peptide induced BTB disruption by *in vivo* BTB integrity assay. In control rat testes, FITC-inulin (green fluorescence) administered to rats at the jugular vein was found to be excluded from entering the adluminal compartment of the epithelium (Fig. 5A), consistent with the presence of a functional BTB located near the basement membrane. In rats treated with F5-peptide at dose of 320 μg/testis, BTB integrity was compromised (Fig. 5B), even though this damage was not as severe as the CdCl_2_-induced irreversible BTB disruption^7^. However, when testes were pre-transfected with Slc15a1 siRNA duplexes to specifically suppress Slc15a1 expression, F5-peptide was found to lose its capability to induce BTB damage (Fig. 5D). Testes receiving both non-targeting siRNA duplexes and F5-peptide showed no difference with those treated with F5-peptide alone (Fig. 5C). Findings shown in Fig. 5A–D were further analyzed semi-quantitatively by comparing the distance traveled by FITC-inulin beyond the BTB near the basement membrane (D_*FITC*_) versus the radius of the seminiferous tubule (D_*STr*_) (Fig. 5E).

### Slc15a1 knockdown blocks F5-peptide induced impairment of spermatogenesis *in vivo*

**Figure 6:**
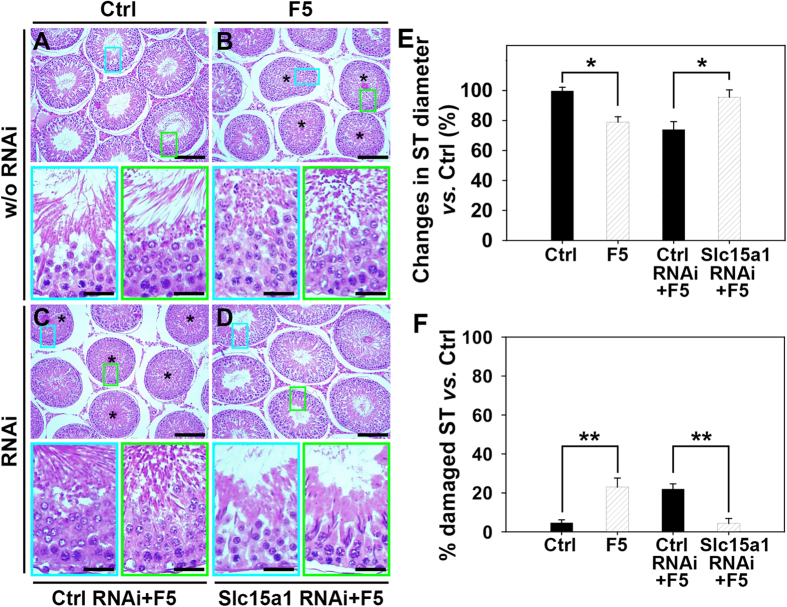
Effect of Slc15a1 knockdown on F5-peptide induced impairment of spermatogenesis and germ cell loss in rat testes. (**A**) Representative photographs of paraffin sections of testes stained with hematoxylin and eosin. Results of HE staining were shown in normal testis (Ctrl, **A**), testis treated with F5-peptide at 320 μg/testis (F5, **B**), or testis pre-transfected with non-targeting control siRNA (Ctrl RNAi+F5, **C**) or Slc15a1 siRNA (Slc15a1 RNAi+F5, **D**) of a group of adult rats (~300–350 gm b.w.) with n = 3 rats in each treatment group. Scale bar in ***A–D*** = 150 μm. The “blue” and “green” encircled micrographs are magnified images of the corresponding boxed areas of the lower magnification, bar = 50 μm. (**E**,**F**) Morphometric changes of seminiferous tubules (ST) (*n* = 200 tubules from 3 rat testes). The shrinkage of tubules indicated by changes in ST diameter (the average ST diameter in treatment group *vs.* that in its corresponding control) (**E**), and the percentage of damaged ST number manifested by germ cell exfoliation (annotated by asterisks in tubules seen in **B**,**C**) in treatment group *vs.* that in its corresponding control (**F**) are summarized in the bar graphs (**E**,**F**), respectively. ^*^P < 0.05, ^**^P < 0.01 when compared to normal control rats.

We then moved to examine the role of Slc15a1 in F5-peptide induced impairment of spermatogenesis in rats. Synthetic F5-peptide at 320 μg/testis was administered via intratesticular injection, and histological analysis was performed following hematoxylin and eosin staining using cross-sections of paraffin-embedded testes (Fig. 6A–D). Histological analysis of these testes revealed that synthetic F5-peptide caused germ cell depletion from the epithelium, mostly on elongated, elongating or round spermatids and some late spermatocytes in treatment (Fig. 6B) *vs*. control (Fig. 6A). However, when testes were pre-transfected with Slc15a1 siRNA duplexes to specifically suppress Slc15a1 expression, F5-peptide was found to lose its capability to induce germ cell loss (Fig. 6D) *vs*. that in non-targeting siRNA pre-treated testes (Fig. 6C). Above treatments did not elicit statistically significant changes in testis weight since cell adhesion was affected in only ~20% of the tubules examined (Fig. 6E,F). The tubule diameter was reduced by ~20% (Fig. 6E,F) in ~20% of the tubules randomly scored, consistent with the observations shown in Fig. 6A–D.

## Discussion

The primary aim of this study is to determine whether Slc15a1 is a potential carrier involved in the transport of synthetic F5-peptide into the seminiferous epithelium where this peptide exerts its physiological functions such as inducing BTB disruption and germ cell loss^7^. RT-PCR (Fig. 1A) and Western blot (Fig. 1B) results revealed Slc15a1 was expressed by the testes, in accordance with earlier reports^12^,^13^. However, Slc15a1 was also detectable in germ cells, myoid cells and Leydig cells, but not in Sertoli cells (Fig. 1A,B), which was in contrast with the distribution pattern of P-gp and MRP1, two best-studied drug transporters in the testes where they were predominantly expressed by Sertoli cells^13^,^18^, suggesting their distinctive physiological functions in the testes. In order to obtain a more visual understanding on Slc15a1’s distribution in the testis at the cellular level, an enzyme immunohistochemistry was performed and results illustrated that Slc15a1 apparently located at tunica propria (*i.e.*, peritubular myoid cells), interstitium (*i.e.*, Leydig cells) and blood vessel endothelium (Fig. 2B–D), relatively weaker signals were also detected in the nuclei of germ cells at all stages of seminiferous epithelial cycle (Fig. 2B–D). Immunofluorescence microscopy further validated above observation and again confirmed the absence of Slc15a1 at the BTB since it failed to co-localize with BTB constituent marker ZO-1 or N-cadherin (Fig. 2F–G). This is the first report on Slc15a1’s precise cellular localization in testes and is the basis for the following functional studies.

Tunica propria functions as one of the major protective barriers in the testes that defends the entrance of harmful or unwanted substances^19^. It is worth noting that Slc15a1 predominantly localized to peritubular myoid cells (Fig. 2B–D), this is interesting because in rodents peritubular myoid cells are known to contribute, at least to some extent, to BTB function^19^,^20^. As such, it is possible that myoid cells also function in the protection of seminiferous epithelium against unwanted agents and drugs. Regarding Slc15a1 as an influx peptide transporter and its strong tunica propria localization surrounding seminiferous tubules, we assumed that Slc15a1 might be involved in transporting exogenous substances, such as the synthetic F5-peptide into seminiferous epithelium where it took further actions. To validate this hypothesis, we next performed *in vivo Slc15a1* gene silencing in rat testes to examine whether knockdown of Slc15a1 would prevent or promote F5-peptide induced impairment of spermatogenesis.

Following the local injection of synthetic F5-peptide into rat testes, no significant change on the steady-state protein level of Slc15a1 was observed up to 5 days post-treatment (Fig. 3). Generally speaking, the up-regulation of transporter proteins such as P-gp^13^ or oatp3^21^ in the testis following drug stimulation is often observed and results in the acceleration of substrate efflux or influx. However, the maintenance of steady-state level of Slc15a1 upon F5 stimulation does not necessarily mean that Slc15a1 could not be a potential transporter for F5-peptide, since the expression level of transporter proteins cannot solely reflect their transport capacity. In addition, F5-peptide administration also did not significantly affect the result of *Slc15a1* knockdown (Fig. 4B). When *Slc15a1* was pre-silenced in rat testis by *in vivo* RNAi, F5 failed to induce any changes as the testis showed the same normal BTB integrity (Fig. 5D) and morphology (Fig. 6D) as the untreated control testis (Figs 5A and 6A). The disruption of BTB integrity (Fig. 5C), germ cell loss, shrinkage of seminiferous tubule, and impairment of spermatogenesis (Fig. 6C) were all observed in non-targeting siRNA-pretreated testis, suggesting that a unique mechanism involving Slc15a1 is in place to transport F5-peptide into the seminiferous epithelium and exert function beyond the BTB site. The presence of influx transporter at tunica propria for F5-peptide well explains a previous question that why administering F5-peptide basally to rat testes *in vivo* would take effects since the receptor for F5-peptide if exists would presumably be concentrated above the BTB^7^. As the anti-F5 antibody or the fluorescence-tagged F5-peptide is currently unavailable commercially, we are making every effort to prepare, purify and test an antibody for F5-peptide with high specificity and purity. Investigation on the uptake of F5-peptide into myoid cells or basal compartment with the use of an anti-F5-peptide antibody will strongly support above hypothesis.

Protein and long peptide normally need to be hydrolyzed into short fragments, such as di- or tri-peptide for the intestinal absorption via Slc15a1. While in the testis our results show that the intact F5-peptide, 50 amino acids in length, could also be a substrate and transported via Slc15a1. This is not unpredictable since oligopeptides serve as nutrient substance in intestinal tract, while F5-peptide functions most probably as a signaling molecule in the testis and thus it does not need to and even cannot be shortened. The present study has revealed that the efficacy via local administration of F5-peptide in the testis is only ~20% (Figs 5E and 6E,F), the highest up to ~40%^7^. Thus, the gene modification or the protein spatial re-conformation of Slc15a1 to produce binding sites with higher affinity might be an improved delivery method for F5-peptide into the seminiferous epithelium where it functions as a potential male contraceptive.

At this point, we also question whether Slc15a1 is the only carrier for transporting F5-peptide into the seminiferous epithelium. We are taking it upon ourselves in a separate but closely related study, to investigate the involvement of Slc15a1 in F5-peptide induced BTB dynamic changes in cultured Sertoli cells, a different system from the *in vivo* one used in this study because no germ cells are present and F5-peptide is in fact treated apically instead of basally *in vivo*. We are particularly interested to determine whether the overexpression of Slc15a1 in Sertoli cells can promote the transient open of the permeability barrier by facilitating F5-peptide entry. We anticipate that further studies will provide new and important insights on the dual role of Slc15a1 in nutrition absorption and cell junction dynamics. Additional studies are now warranted to investigate if Slc15a1 can be modified into a more suitable carrier with high transport capacity for F5-peptide, a potential male contraceptive, to assist it to enter the seminiferous epithelium and exert functions. We expect that some of these questions will be addressed in the near future.

## Materials and Methods

### Animals

The animal procedure described in this study was approved by the Fourth Military Medical University Institutional Animal Use and Care Committee (Xi’an, China). Adult male Sprague-Dawley (SD) rats were obtained from Animal Center in the Fourth Military Medical University. Studies were performed in accordance with the Guide for the Care and Use of Laboratory Animals defined by National Research Council (the National Academies, Eighth Edition, 2011). All rats were maintained at 22 °C with a 12/12-light/dark cycle and received lab chow and water ad libitum.

### Testicular cell isolation

Primary Sertoli cells were isolated from the testes of 20-day-old SD rats as described previously^22^. It is noted that Sertoli cells isolated from rats at 20 days of age are fully differentiated and cease to divide^23^. Sertoli cells were then plated on Matrigel (BD bioscience, San Jose, CA)-coated dishes at 0.5 × 10^6^ cells/cm^2^ and cultured in serum-free F12/DMEM (Sigma-Aldrich, St. Louis, MO) supplemented with growth factors, bacitracin, and gentamicin at 35 °C in a humidified atmosphere with 95% air/5% CO2 (v/v)^22^. Two days thereafter, Sertoli cells were subjected to hypotonic treatment to lyse residual germ cells, as described previously^24^, so that these cultures had a Sertoli cell purity >98% and were contaminated with negligible Leydig and germ cells. Sertoli cells were then harvested for lysate preparation.

Total germ cells were isolated from testes of adult SD rats (∼250 g b. w./each) by a mechanical procedure without using trypsin and were not subjected to the glass-wool filtration step so that elongating/elongated spermatids were retained in the germ cell population^25^. Total germ cells were plated on 100-mm dishes at 2.5 × 10^6^ cells/ml and cultured in F12/DMEM supplemented with 2 mM sodium pyruvate and 6 mM sodium DL-lactate as described previously^25^. Germ cells were harvested for lysate preparation after ∼16 h in culture at 35 °C with a viability of greater than 95% by the erythrosine red dye exclusion test, as described previously^26^.

Leydig cells were isolated from adult SD rats and purified by discontinuous Percoll gradient as previously described^27^, while peritubular myoid cells were isolated from 20-day-old SD rats by sequential enzymatic digestion as described in^28^.

### RNA extraction and RT-PCR

For RNA extraction, testes, Sertoli cells, and germ cells were lysed in TRIzol reagent (Invitrogen, Carlsbad, CA) according to the manufacturer’s instructions. The extracted total RNA was reverse transcribed to cDNA using M-MLV reverse transcriptase (Promega, Madison, WI). The cDNAs of *Slc15a1* and *S-16* genes were amplified by PCR using GoTaq DNA polymerase (Promega) with specific primer pairs (Table 1). The authenticity of PCR products was verified by DNA sequencing.

### Lysate preparation and immunoblotting

Lysates of testes, Sertoli cells, and germ cells were prepared in IP lysis buffer: 50 mM Tris, pH 7.4 at 22 °C, containing 150 mM NaCl, 2 mM EGTA, 10% glycerol (v/v), 1% Nonidet P-40 (v/v), and freshly supplemented with protease inhibitor cocktail (Sigma-Aldrich) and phosphatase inhibitor cocktail 1 and cocktail 2 (Sigma-Aldrich) using a lysis buffer:inhibitor ratio (v/v) at 100:1. Immunoblotting was performed as described previously^13^ with a rabbit anti-Slc15a1 primary antibody (Table 2) using ~50 μg protein lysates per sample for SDS-PAGE. Equal protein loading was assessed by stripping blots and re-probed with a goat anti-β-actin antibody (Table 2).

### Immunohistochemistry and immunofluorescent staining

Frozen testis sections at 7-μm thickness obtained in a cryostat at −20 °C were fixed with 4% paraformaldehyde (w/v) in PBS for 10 min at room temperature and permeabilized with 0.1% Triton X-100 (v/v) in PBS for 10 min at room temperature. Immunohistochemistry was carried out with a AEC Detection System kit (Millipore, Billerica, MA) according to the manufacturer’s instructions. For immunofluorescent staining, sections were blocked with 1% BSA (w/v) in PBS, to be followed by an overnight incubation of primary antibodies (Table 2) at 1:50 dilution in PBS, and then a 30-min incubation of Alexa Fluor-conjugated (Alexa Fluor 488, green fluorescence; Alexa Fluor 555, red fluorescence) goat secondary antibodies (Invitrogen) at 1:200 dilution in PBS. Sections were mounted in ProLong Gold antifade reagent with DAPI (Invitrogen). To eliminate interexperimental variations, all cross sections of testes within a treatment group were mounted onto the same microscope slides and processed simultaneously. All staining experiments were repeated three times using different animals.

### Treatment of rat testes with synthetic F5-peptide

Adult rats weighing ~250 g body weight (n = 3 rats per time point in each treatment group) received a single dose of synthetic F5-peptide (peptide information is available in^7^) at 320 μg per testis *via* direct intratesticular injection using a 28-gauge needle in a final injection volume of ~200 μl (in 0.9% saline) as described earlier^29^,^30^. This concentration of synthetic F5-peptide was selected based on the *in vitro* results as reported earlier^7^. Rats were euthanized by CO_2_ asphyxiation on selective time points and testes were removed. In selected experiments, testes were either fixed in Bouin’s fixative to be used for paraffin embedding and sectioning for hematoxylin and eosin staining for histological analysis as described^31^, or snap frozen in liquid nitrogen and stored at −80 °C until used to obtain testis lysates for immunofluorescence or immunoblotting.

### Slc15a1 RNAi in adult rat testes *in vivo*

Adult male Sprague-Dawley rats (∼250 g b. w./each) were treated with non-targeting control or Slc15a1-specific siRNA transfection mix *via* intratesticular injection using a 28-gauge needle, as described previously^29^,^30^. To silence Slc15a1, four different siRNA sequences (J-095173-21: 5′-CAGCAGAGAUCGAGGCACAtt; J-095173-22: 5′-GCAUCAUAUUUGCCAUUAUtt; J-095173-23: 5′-ACAAACAGUGGGCUGAGUAtt; J-095173-24: 5′-CGUUGGAACCUGUCUCACAtt; Thermo Scientific) were analyzed and individually used. The ON-TARGETplus Non-targeting Pool (D-001810-10, Thermo Scientific) was used as control. Each transfection mix (final volume ∼200 μl) consisted of 100 nM siRNA duplexes and 7.5 μl Ribojuice siRNA transfection reagent (Invitrogen) suspended in 190 μl Opti-MEM (Invitrogen). The volume of each testis was assumed to be ∼1.6 ml. Different pilot experiments were performed to assess the optimal ratio of Opti-MEM to Ribojuice. Control and Slc15a1 RNAi transfection mix were administered to a different testis in the same rat, so that each animal consisted of a control and a treated testis. Rats were terminated for western blotting analysis, histological analysis using paraffin sections, or *in vivo* BTB integrity assay at least two days after RNAi treatment.

### Hematoxylin and eosin staining

Paraffin sections at 7-μm thickness were obtained from Bouin’s fixed testes embedded in paraffin. After dewaxing with xylene, sections were stained with Hematoxylin 7211 for 3 min, to be followed by 1-min incubation of Clarifier 1 and Bluing Reagent, and then stained with Eosin-Y for 30 s (reagents from Richard-Allan Scientific, Richland, MI). Results were analyzed in conjunction with 3 sets of frozen testis sections stained with DAPI in separate experiments to quantify the percentage of tubules displaying germ cell loss from the seminiferous epithelium.

### BTB integrity assay

Each rat was under anesthesia with ketamine HCl (60 mg/kg b. w.) together with xylazine (10 mg/kg b. w.) administered i.m. Thereafter, a small incision on the skin over the jugular vein was made, and the vein was carefully exposed. About 1.5 mg FITC-conjugated inulin (Mr = 4.6 kD) (Sigma-Aldrich) in 300 μl PBS was administered into the jugular vein using a 28-gauge needle. About 30 min thereafter, rats were euthanized by CO_2_ asphyxiation. The integrity of the BTB was assessed by its ability to block inulin-FITC from entering the adluminal compartment, as visualized by fluorescent microscopy in frozen testis sections. Damaged tubules were defined herein by the penetration of green fluorescence across at least 3 layers of cells behind the BTB toward the tubule lumen.

### Statistical analysis

Each experiment reported herein was repeated at least 3 times, excluding pilot experiments. Statistical analysis was performed using the GB-STAT software package (version 7.0; Dynamic Microsystems, Silver Spring, MD). For multiple comparisons, 1-way ANOVA was performed and followed by Dunnett’s test. This thus compared multiple treatment groups against the control. In selected experiments, Student’s *t* test was used for paired comparisons.

## Additional Information

**How to cite this article**: Su, L. *et al.* Slc15a1 is involved in the transport of synthetic F5-peptide into the seminiferous epithelium in adult rat testes. *Sci. Rep.* **5**, 16271; doi: 10.1038/srep16271 (2015).

## Figures and Tables

**Table 1:** Primers used for RT-PCR in this study.

Gene	Primer sequence	Orientation	Position	Length (bp)	A.T. (°C)	Cycle no.	GenBank^®^accession no.
***Slc15a1***	5′- CTCTGCTACCTGACTCCAA-3′	sense	184–202	223	53	25	NM_057121
	5′- TACCAAGGGCTATCAGGG-3′	anti-sense	389–406				
***S-16***	5′- TCCGCTGCAGTCCGTTCAAGTCTT-3′	sense	15–38	385	53	25	XM_341815
	5′- GCCAAACTTCTTGGTTTCGCAGCG-3′	anti-sense	376–399				

A.T., annealing temperature.

**Table 2:** Summary of primary antibodies used in this study.

Antigen	Catalog no.	Immunogen	Host	Vendor	Dilution		
					IB	IHC	IF
**Slc15a1**	LS-C18855	Synthetic peptide corresponding to human SLC15A1;	Rabbit	Lifespan biosciences	1:1000	1:50	1:50
	sc-20653	Epitope corresponding to amino acids 366-600 of PEPT1 of human origin	Rabbit	Santa cruz biotechnology	1:200		
**ZO-1**	61-7300	Fusion protein corresponding to amino acid residues 463-1109 of human ZO-1	Rabbit	Zymed/Invitrogen			1:50
**N-Cadherin**	sc-7939	Amino acid residues 450-512 of human N-cadherin	Rabbit	Santa cruz biotechnology			1:50
**Actin**	sc-1616	Peptide mapping of C-terminus of human actin	Goat	Santa cruz biotechnology	1:200		

IB, immunoblotting; IHC, immunohistochemistry; IF, immunofluorescence.
